# Protein vaccine NVX-CoV2373 elicits functional T cell immunity

**DOI:** 10.1172/JCI163614

**Published:** 2022-10-03

**Authors:** Pengcheng Zhou

**Affiliations:** Laboratory of Molecular Immunology, The Rockefeller University, New York, New York, USA.

## Abstract

The SARS-CoV-2 vaccine NVX-CoV2373 is a protein-based vaccine that might circumvent the difficulties in distributing mRNA vaccines to regions with limited access to cold-chain and refrigeration. However, the NVX-CoV2373–induced T cell and antibody responses remain poorly understood. In this issue of the *JCI*, Moderbacher et al. characterized SARS-CoV-2–specific CD4^+^ and CD8^+^ T cell responses elicited by one or two doses of NVX-CoV2373 in individuals enrolled in a phase I/IIa trial. Substantially increased spike-specific CD4^+^ and T follicular helper cells were found after the first or second vaccine dose, with some individuals developing a modest spike-specific CD8^+^ T cell response. Correlation analysis revealed an association between spike-specific CD4^+^ T cells and neutralizing antibody titers. Notably, preexisting T cell immunity showed negligible effects on NVX-CoV2373–induced T cell responses. These findings indicate that the protein-based vaccine NVX-CoV2373 induces robust T cell immunity capable of recognizing SARS-CoV-2 antigens and supporting humoral immune responses.

## Long-term protection

Upon vaccination, individuals develop cellular and humoral immune responses that provide long-term protection against antigen reexposure. This process requires the generation of functional CD4^+^ T cells, CD8^+^ T cells, and B cells that recognize antigens delivered by a vaccine. Evaluating the magnitude, longevity, and antigen recognition of T cell and neutralizing antibody responses is the gold standard to understand the quality of the vaccine-elicited immune response. Examining these immunological parameters also helps understand the mechanisms of action and the duration of a vaccine-induced protection, which assists the formulation of future vaccine strategies to combat a pathogen. Vaccine platforms such as mRNA vaccines (Pfizer/BioNTech BNT162b2, Moderna mRNA-1273) and a viral vector–based vaccine (AstraZeneca, Janssen Ad26.COV2.S) efficiently induce these functional immune cells upon administration in humans (1).

**Figure 1:**
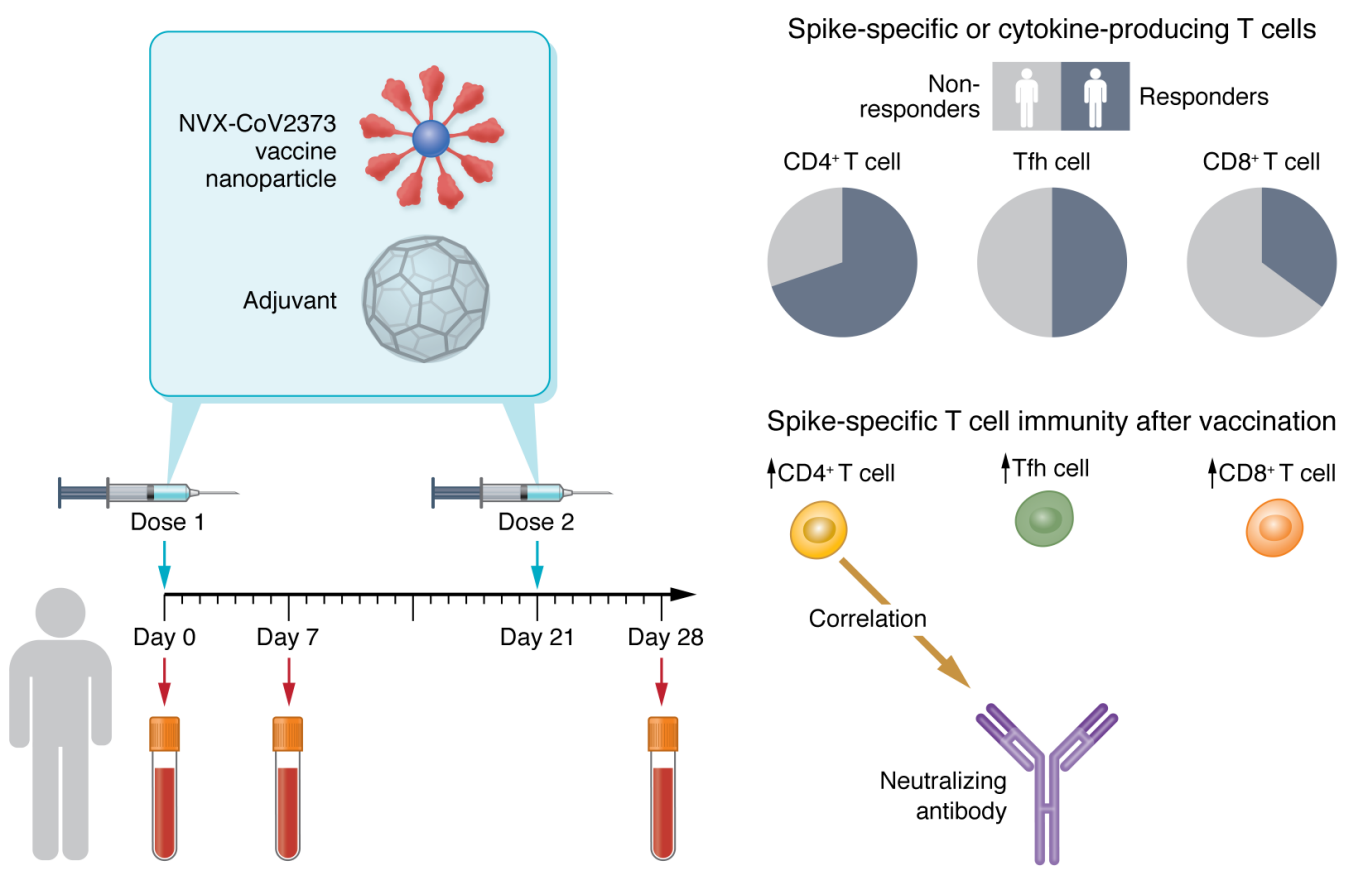
T cell immune response in individuals who received NVX-CoV2373 vaccine. Individuals who received one or two doses of the protein-based NVX-CoV2373 vaccine exhibited increased levels of SARS-CoV-2 spike–specific CD4^+^ T cells, circulating Tfh cells, CD8^+^ T cells, and serum neutralizing antibodies. Spike-specific CD4^+^ T cell frequency correlated with the SARS-CoV-2–neutralizing antibody titers. Spike-specific CD4^+^ T cell response was present in most of the individuals who received one or two doses of NVX-CoV2373 vaccine, while a smaller proportion of vaccinees displayed spike-specific circulating Tfh cells and spike-specific CD8^+^ T cells.

NVX-CoV2373 is a SARS-CoV-2 vaccine that received approval by the Food and Drug Administration (FDA) for emergency use in individuals aged 18 and above. It has been shown to be safe and effective in providing protection against severe COVID-19 (2). This vaccine applies recombinant spike protein trimers from the ancestral SARS-CoV-2 strain as assembled nanoparticles that are mixed with Matrix-M adjuvant to induce an immune response (Figure 1). Different from mRNA vaccines that require stringent cold-chain for deployment and storage, protein-based NVX-CoV2373 is stable at 2°C–8°C, making it manageable to distribute in regions where refrigeration is limited. Amid the emerging evidence on the safety and efficacy of the NVX-CoV2373 vaccine from experimental trials, little is known about the human immune response following NVX-CoV2373 vaccination. In this issue of the *JCI*, Moderbacher et al. set out to unravel this question by utilizing the samples from individuals in a phase I/IIa clinical trial who received 5 μg of NVX-CoV2373 protein together with Matrix-M adjuvant (3, 4).

## SARS-CoV-2–specific CD4^+^ T cell and T follicular helper cells

Antigen-activated CD4^+^ T cells provide help to antibody and cytotoxic immune responses against pathogens (5). By isolating peripheral blood mononuclear cells (PBMCs) from vaccinated donors, Moderbacher et al. found that the NVX-CoV2373 vaccine substantially increased the frequency of spike-specific CD4^+^ T cells by day 7 after the first and second vaccinations compared with baseline, as measured by an ex vivo activation induced marker (AIM) assay with SARS-CoV-2 spike peptide stimulation (3). Although a larger proportion of individuals showed detectable SARS-CoV-2 spike–specific CD4^+^ T cells after the second dose, no differences were found in the magnitude of these T cells between two doses of NVX-CoV2373 vaccine. T follicular helper (Tfh) cells are specialized CD4^+^ T cells that provide help to the antibody-based immunity in germinal centers (6–9). Increasing evidence shows that Tfh cells play a major role in mounting protective antibody responses in infection and vaccination against SARS-CoV-2 (10–15). Importantly, the authors observed that nearly half of the individuals showed increased spike-specific circulating Tfh (cTfh) cells after two doses of NVX-CoV2373 vaccine, suggesting that NVX-CoV2373–induced CD4^+^ T cells might be capable of supporting an antibody response.

Activated CD4^+^ T cells produce cytokines to coordinate immune responses (16). To evaluate the functional cytokine profiles of spike-specific CD4^+^ T cells and Tfh cells, Moderbacher et al. performed intracellular cytokine staining of peptide-stimulated CD4^+^ T cells. They found that spike-specific IFN-γ, TNF-α, and IL-2 production in CD4^+^ and Tfh cells was markedly increased 7 days after the first vaccination with NVX-CoV2373, accompanied with further increases after the second vaccine dose. Further experiments showed that one or two doses of NVX-CoV2373 substantially increased the polyfunctional spike-specific CD4^+^ T cells, as determined by expression of at least two effector molecules, including intracellular CD40L, GzmB, IFN-γ, TNF-α, and IL-2 within the same CD4^+^ T cells (3). In parallel, circulating Tfh cell cytokine responses largely mirrored the overall CD4^+^ T cell response, with a polarization toward Th1 cytokine production (3). These findings suggest that NVX-CoV2373 elicits functional SARS-CoV-2–specific CD4^+^ T cells (Figure 1).

## SARS-CoV-2–specific CD8^+^ T cells

Conventionally, protein vaccines do not have a record as good inducers of CD8^+^ T cell responses, as compared with live-attenuated or inactivated vaccines (17). With pathogens that are less vulnerable to antibody-mediated protection, such as *Listeria monocytogenes*, or are highly mutating, such as flu virus, CD8^+^ T cells play a critical role in providing protective immunity (18). mRNA vaccines and natural infections induce robust memory CD8^+^ T cell responses that cross-recognize SARS-CoV-2 variants, including Omicron (19). It was encouraging that a substantial proportion of vaccinees displayed spike-specific CD8^+^ (CD69^+^4-1BB^+^) T cells after the first and second vaccinations with NVX-CoV2373 compared with baseline (3). Moreover, a slightly smaller proportion of volunteers showed functional cytokine production (IFN-γ, TNF-α, and IL-2) by spike-specific CD8^+^ T cells, while no further increases in such production were found following the second vaccine dose (3). The overall percentage of AIM^+^ or cytokine^+^ spike-specific CD8^+^ responders to one or two doses of NVX-CoV2373 was below 40% (Figure 1). Given the fact that 45%–80% of individuals who received one or two doses of mRNA vaccine or viral vector–based vaccine developed spike-specific CD8^+^ T cells, this response in NVX-CoV2373 vaccinees is modest (20). It would be interesting to see whether a third booster of NVX-CoV2373 would increase the proportion of spike-specific CD8^+^ responders. Further studies are required to test different adjuvants that might enhance the protein-vaccine-induced CD8^+^ T cell response.

## Association between T cell and antibody responses

How T cell immunity acts collaboratively with B cells and antibodies is a high-priority question in understanding the mechanisms and dynamics of a vaccine-induced immune response. Tfh cells, the gatekeepers of germinal center responses, are known to help select mature B cells for high-affinity antibodies, and represent a substantial proportion of total antigen-specific (AIM^+^) CD4^+^ T cells (21). This frequency is around 47% in recovered COVID-19 patients and over 20% in naive individuals who received one dose of mRNA vaccine (21). It is tempting to speculate that antigen-specific Tfh and CD4^+^ T cells should associate with antibody responses in vaccinees with NVX-CoV2373. Interestingly, Moderbacher et al. found that spike-specific CD4^+^ T cells, but not spike-specific cTfh cell frequencies, were associated with neutralizing antibodies after both the first and second vaccination with NVX-CoV2373 (3). One explanation for this could be the phenotypic and functional discrepancies between the cTfh cells and germinal center Tfh cells in lymph nodes. Indeed, germinal center Tfh cells were found markedly correlated with antibody titers, while Tfh cells in PBMCs usually exhibited borderline association with antibodies in SARS-CoV-2 infection and vaccination (12, 15). Notwithstanding, a study with a larger sample size might provide better clues on relationships between cTfh cells and antibody response following NVX-CoV2373 vaccination. Notably, a population of CXCR5^–^PD-1^+^CD4^+^ T cells was found to be superior in providing B cell help in autoimmune disease (22). These peripheral helper T (Tph) cells might overrepresent the total spike-specific CD4^+^ T cells that correlated with antibodies following NVX-CoV2373 vaccine. How Tph cells participate in the cellular and humoral anti–SARS-CoV-2 response could be of interest in future studies.

## Preexisting T cell immunity

Ten donors showed detectable SARS-CoV-2 spike–specific CD4^+^ T cell responses before receiving the NVX-CoV2373 vaccine (3). Moderbacher and colleagues set out to examine how this preexisting T cell immunity might shape NVX-CoV2373–induced vaccine responses. Having established that the donors had low possibilities of previous antigen exposure to SARS-CoV-2 based on the low local SARS-CoV-2 seroprevalence at the time of recruitment, the authors found that preexisting T cells had no effects on the magnitude of the spike-specific CD4^+^ T cell response after the first or second vaccination. Similar negligible influences were found with spike-specific cTfh cells and CD8^+^ T cells and their cytokine production (3). Notably, the sample size of this study was too small to make a clear conclusion. Moreover, it will be interesting to compare the differences between mRNA and protein vaccines in their mechanisms of priming or boosting T cell and B cell responses, as inconsistent results were found across different vaccine platforms on the effects from preexisting immunity (21, 23). The hypothesis posits that preexisting T cell memory, mostly found in CD4^+^ T cells, might either lead to a beneficial and faster neutralizing antibody response upon antigen reexposure (24), or in contrast, restrict the participation of naive T cells in the subsequent immunity due to their TCR specificity and affinity.

## Implications and prospects

This clinical study by Moderbacher et al. indicates that protein-based vaccine NVX-CoV2373 induced functional human antibodies as well as CD4^+^ T cell and relatively modest CD8^+^ T cell immunity against SARS-CoV-2 infection. Other topics that remain to be explored include the evaluation of memory B cell and long-lived plasma cell responses and how germinal center responses would evolve following NVX-CoV2373 vaccination. It appears that two doses of mRNA vaccine induce persistent germinal center B cells for at least 6 months (25). It would also be interesting to follow the NVX-CoV2373–elicited T cells longitudinally and examine how memory CD4^+^ and CD8^+^ T cells persist to clonally expand or contract. Although three doses of mRNA vaccine effectively prevents the severe disease from Omicron subvariant infection, it becomes apparent that variants of concern (VOCs) such as BA.4 and BA.5 show higher neutralization escape than the ancestral strains of SARS-CoV-2 (26, 27). To what extent the VOCs might escape from NVX-CoV2373–induced immune protection requires urgent investigation. Booster combinations with a homologous NVX-CoV2373 vaccine or heterologous vaccines from other platforms are also of interest and might extend different breadth and longevity of protective immune responses to SARS-CoV-2. Studies that confirm these data in larger cohorts are eagerly awaited.

## Version 1 10/03/2022

Electronic publication
